# Featured Reviews in Organic Chemistry

**DOI:** 10.3390/molecules28165975

**Published:** 2023-08-09

**Authors:** Roman Dembinski, Vadim Soloshonok

**Affiliations:** 1 Centre of Molecular and Macromolecular Studies, Polish Academy of Sciences, Sienkiewicza 112, 90-363 Łódź, Poland; 2 Department of Chemistry, Oakland University, 146 Library Dr., Rochester, MI 48309-4479, USA; 3 Department of Organic Chemistry I, Faculty of Chemistry, University of the Basque Country (UPV/EHU), Paseo Manuel Lardizábal 3, 20018 San Sebastián, Spain; 4 IKERBASQUE, Basque Foundation for Science, Alameda Urquijo 36-5, Plaza Bizkaia, 48011 Bilbao, Spain

The field of Organic Chemistry represents one of the most traditional areas of chemistry that has delivered many benefits to the community of chemists. This Special Issue of *Molecules* is a compilation of the most recent innovative developments in organic chemistry, synthesis, etc. The collected articles provide summaries and assessments of a range of pharmaceutical expansions, organic and bioorganic studies, methodologies, and reactions, as well as critical views and perspectives with regard to future developments. The Editors were fortunate to assemble a compendium of articles, written by prominent authors, which represent the broad field of organic and related chemistry. 

It is worth pointing out the excellent coverage of the following areas:

*Halogen-Functionalization*

In the review covering recent advances on the halo- and cyano-trifluoromethylation of alkenes and alkynes, a team of authors led by Escorihuela, Han, and Fustero discuss the most significant contributions during the last decade. The reactions reviewed in this work include chloro-, bromo-, iodo-, fluoro-, and cyano-trifluoromethylation of alkenes and alkynes, providing a perspective for functionalization of unsaturated carbon–carbon multiple bonds [1].

*Pharmaceutical Advances During 2021/2022 (with a Focus on Halogen Containing Structures)*

The strategic fluorination of oxidatively vulnerable sites in bioactive compounds is a relatively recent, widely used approach allowing to modulate the stability, bio-absorption, and overall efficiency of pharmaceuticals. In parallel, natural and tailor-made amino acids are traditionally used as basic scaffolds for the development of bioactive molecules. A team lead by Dhawan, Zhang, Han, and Soloshonok offers a summary of new approved drugs appearing in the pharmaceutical market in 2022. This review article emphasizes these general trends featured in recently approved pharmaceutical drugs. The article features fragments of tailor-made amino acids and fluorine [2].

The article by Benedetto Tiz, Santi, et al. compiles halogen-containing drugs approved by the FDA in 2021, providing an overview on their syntheses and pharmaceutical uses [3]. Selected molecules contain at least one covalently bound halogen atom. The role of halogens (fluorine and chlorine in particular) in the preparation of drugs for the treatment of several maladies such as viral infections, several types of cancer, cardiovascular disease, multiple sclerosis, migraine, and inflammatory diseases such as vasculitis is indicated.

*Bio-Related Molecules*

The review of Török, B. and Török, M. surveys the major structural features in various groups of small molecules that are considered to be antioxidants, including natural and synthetic compounds alike [4]. Recent advances in the strategic modification of known small molecule antioxidants are also described. The emphasis is placed on changing major physicochemical parameters, including log *p*, bond dissociation energy, ionization potential, and others, which results in improved antioxidant activity.

The article by Janczewski covers the synthesis and biological activity of sulforaphane and its bifunctional analogs [5]. The methods of synthesis, as well as sulforaphane natural or synthetic bifunctional analogues with various functional groups, are discussed, and their biological activity is also summarized.

A critical discussion of the data in the literature reporting on the preparation of substituted γ-aminobutyric acid (GABA) derivatives using the Michael addition reaction as a key synthetic transformation is provided by Fustero, Sorochinsky, et al. [6]. GABA represents one of the most prolific structural units which is widely used in the design of modern pharmaceuticals. In this article, special attention is paid to asymmetric methods featuring synthetically useful stereochemical outcomes and operational simplicity.

*Radical Reactions*

Radical reactions are powerful in terms of creating carbon–carbon and carbon–heteroatom bonds. Zhang’s et al. review describes one-pot radical reactions embedded into cascade transformations to assemble cyclic skeletons with two new functional groups. Those sequential radical addition and cyclization reactions are both synthetically and operationally efficient. Summarized in the article is the recent development of reactions involving radical addition and cyclization of dienes, diynes, enynes, as well as arene-bridged and arene-terminated compounds for the preparation and difunctionalization of cyclic compounds [7]. Reactions carried out with radical initiators, transition metal-catalysis, photoredox, and electrochemical conditions are included.

It is essential to introduce appropriate functional groups at appropriate positions in molecules thus the highly selective preparation of multiple functional groups is considered synthetically important. The article by Yamamoto and Ogawa focuses on radical reactions with high functional group selectivity. This study overviews the recent progress in practical methods for the simultaneous introduction of multiple functional groups and propose future synthetic strategies that emphasize the recycling and environmental impact [8]. Highlighted is the metal-free one-pot multi-functionalization of unsaturated compounds with interelement compounds by radical processes.

*Methodologies*

The progress in the construction of C-C and C-heteroatom bonds using alcohols as acyl precursors was brought to this Special Issue by Zhao, Huang, et al. [9]. While the traditional Friedel–Crafts acylation processes work to allow for viable construction of arylketones under harsh acidic conditions, recent progress in the development of acylation methods has focused on new reactivity discovery by exploiting versatile and easily accessible acylating reagents. Among them, alcohols are readily available, exhibit low toxicity, and are naturally abundant feedstocks; thus, they have recently been used as ideal acyl precursors in molecule synthesis for ketones, esters, amides, etc. Recent advances in terms of employing alcohols as unusual acyl sources to form C-C and C-heteroatom bonds, with emphasis on the substrate scope, limitations, and mechanisms, are discussed.

The α-branched aldehydes are especially challenging substrates due to reactivity and selectivity issues. Despite several problems, in the last 15 years, several catalytic approaches for the α-functionalization of prostereogenic α-branched aldehydes that proceed in useful yields and diastereo- and enantioselectivity have been uncovered. Developments including organocatalytic and metal-catalyzed approaches as well as dual catalysis strategies for forging new carbon–carbon and carbon–heteroatom (C-O, N, S, F, Cl, Br, …) bond formations at Cα of the starting aldehyde are the subject of the review authored by the team led by Mielgo and Oiarbide [10]. 

The review summarizing the synthesis of functionalized six-membered-ring azahelicenes, written by Fontana and Bertolotti, aims to provide a survey of the different synthetic methods available to attain this intriguing class of compounds [11]. The synthesis of the helicene can be performed using starting materials that already contain a side group, or the side group can be introduced after the synthesis of the parent structure. As azahelicenes are helicenes bearing one or more nitrogen atom(s) in the molecular framework, parent azahelicenes can be functionalized on carbon atoms by exploiting the presence of the electron-withdrawing nitrogen atom. The quaternary salts derivatives, offering solubility and electronic properties different from those of the parent azahelicenes, are also presented.

*Specific Small Molecules*

Indane-1,3-dione is a versatile building block used in numerous applications. Pigot, Brunel, and Dumur reviewed its synthetic strategies and applications. An overview of the different chemical reactions enabling access to this scaffold and its most common derivatives is offered. The versatility of its structure is evidenced by the record of the different applications in which indane-1,3-dione-based structures have been used [12].

Finally, Wheeler, Török, and Dembinski describe the recent advances in the synthesis of isoquinoline-fused benzimidazoles [13]. This review includes recent developments in the synthesis of these four ring-fused dinitrogen-heterocycles, sorting protocols based on the structural features of the reacting components. Crystallographically characterized benzo[4,5]imidazo[2,1-a]isoquinolines are also summarized, and their geometrical properties are analyzed.

In summary, this unique collection of quality reviews deserves special recognition; thus, we were honored to assemble such a compendium.

The Editors would like to thank all the Authors for selecting this Special Issue of *Molecules* as their platform of presentation and for submitting their excellent work. We would also like to thank the Reviewers, as well as the Editorial Office (especially Freya Li), for their support.
